# Community‐centred disaster recovery: A call to change the narrative

**DOI:** 10.1111/disa.12655

**Published:** 2024-09-04

**Authors:** David Sanderson, Tim Heffernan, Marco DeSisto, Clifford Shearing

**Affiliations:** ^1^ School of Built Environment University of New South Wales Sydney Australia; ^2^ College of Business and Law Royal Melbourne Institute of Technology Australia; ^3^ Faculty of Law and Justice University of New South Wales Sydney Australia

**Keywords:** Australia, community‐centred, disaster, recovery

## Abstract

This paper challenges current approaches to undertaking community‐centred disaster recovery. Community‐centred approaches are widely recognised as ‘the gold standard’ for effective recovery from disasters. Yet, they are rarely applied well enough in practice. Challenges include the ‘authority’ culture of command‐and‐control agencies, the emphasis on discrete recovery time frames, and the reluctance to relinquish centralised control. The paper focuses on people's experiences of community‐centred recovery in New South Wales, Australia, which has experienced severe fires and floods since 2019. We undertook key informant interviews and an online survey to inquire into how community‐centred recovery is enacted. Our work uncovered widespread dissatisfaction with current practices. The paper discusses key themes emerging from the research and ends with a call to change how community‐centred recovery is framed and conducted by responding organisations, to include the underlying causes of vulnerability in recovery, to measure success differently, and to alter the narrative of who ‘owns’ disasters.

## Practitioner points

1. The increasing frequency and severity of disasters highlight the need for a rethink on how disaster recovery is understood and can be better achieved.
2. A key problem lies in top‐down command‐and‐control approaches by responding agencies, which are ill‐suited to supporting community‐centred recovery.
3. Systemic change is needed regarding how disaster recovery is framed, including changing the narrative on who ‘owns’ disaster recovery, measuring success differently, and addressing a ‘them and us’ culture between agencies and communities.

## 1 INTRODUCTION

The widespread consensus is that community‐centred recovery from naturally‐triggered disasters such as fires and floods is ‘the gold standard’. This is for good reason. The evidence points to communities being more resilient before, during, and after disasters, when they are in the driving seat in terms of their own decision‐making (Shariff and Hamidi, 2019). In Australia, as elsewhere, community‐centred recovery is widely adopted by state and national agencies and implementing non‐governmental organisations (NGOs) appear regularly in policy and planning documents (AIDR, 2018; Oloruntoba, Sridharan, and Davison, 2018). However, for policymakers, emergency services, and other service providers, enacting community‐centred approaches that communities themselves value can be hard to do (Vallance, 2015; Syamsidik et al., 2021). Experience from across the world following large‐scale disasters suggests that, very often, communities feel disenfranchised and unheard by the very services that strive to support them. Indeed, many argue that achieving genuine community‐centred recovery remains a pipedream, highlighting a tension between policy, procedure, and practice (Syamsidik et al., 2021).

For the purposes of this paper, we define community‐centred recovery from disaster as ‘approaches that mobilise assets within communities, encourage equity and social connectedness and increase people's control over their lives’.^1^ This definition implies that the agency and voice of affected communities in decision‐making are central to achieving effective recovery. It also includes the proposition that, to be effective, recovery measures ought to improve conditions to reduce vulnerability to future disasters, and not just ‘replace’ what was already in place. This is vital given the escalating frequency and severity of disaster threats, which are predicted only to get worse owing to the challenges of climate change and rapid population growth.

This is underscored by the impacts of disasters both on people's way of life and the financial fallout for countries from the effects of natural hazards. Globally, 2021 proved to be one of the costliest disaster years on record, with USD 280 billion in overall losses (Munich Re, 2022). In Australia, for instance, ‘[a]s well as costing lives and livelihoods, disasters cost Australia $38 billion per year on average, with the cost estimated to reach at least $73 billion per year by 2060’ (NEMA, 2022). The predictions are sobering. Concerning floods, a 2024 report concludes that, by 2030, more than three million homes will have exposure to flooding from rivers, with half a million considered to be high risk (Mallon and Lorenz, 2024).

Against this backdrop, a growing number of humanitarian programme evaluations (Clermont et al., 2011; Sanderson and Ramalingam, 2015; Juillard and Jourdain, 2019) and academic scholarship (Crawford and Morrison, 2021; Dhungana and Curato, 2021) continue to report that formal disaster recovery processes routinely overlook, leave out, or treat as an add‐on the participation and leadership of impacted communities and consultation with them. In this context, we return to the questions of what does community‐centred recovery from disaster mean and, crucially, how can it be achieved?

This paper is in three parts. Part one discusses what is meant by community‐centred disaster recovery. Part two presents a case study of the findings of research (key informant interviews and an online survey) undertaken primarily in New South Wales (NSW), Australia, into the experiences of communities of community‐centred disaster recovery. Part three assesses the findings and ends with a discussion that calls for a systemic rethink of how community‐centred recovery is framed and enacted.

### 1.1 Part one: community‐centred disaster recovery—the gold standard

To understand what locally‐driven resilience means and how it can best be achieved, this section looks to the disaster risk reduction and community development literature to provide a framing for the oft‐used, but under discussed, terms ‘disaster recovery’ and ‘community‐centred’, noting the popularity of both among practitioners and scholars alike.

#### 1.1.1 Disaster recovery

The term ‘disaster recovery’ is traditionally applied to the post‐immediate relief phase of a disaster. There are no precise time frames for relief, recovery, and subsequent phases, although many operational agencies mandate specific time frames, largely for political and budgeting reasons (as discussed below). Despite recovery being an essential process in disaster management, there is a shortage of information on what actually works and across what time frames. Several authors (see, for example, Owen, 2018) have pointed out the lack of peer‐reviewed studies focusing on recovery. As Owen (2018, p. 66) concludes, ‘the bulk of material relating to ‘good recovery’ is in the grey literature, is difficult to find and is not comprehensive. While it is common practice for governments to release reports evaluating specific recovery efforts, there is a sense that these are often sanitised. Candid contributions by all parties, including government employees, are rare’.

**FIGURE 1:**
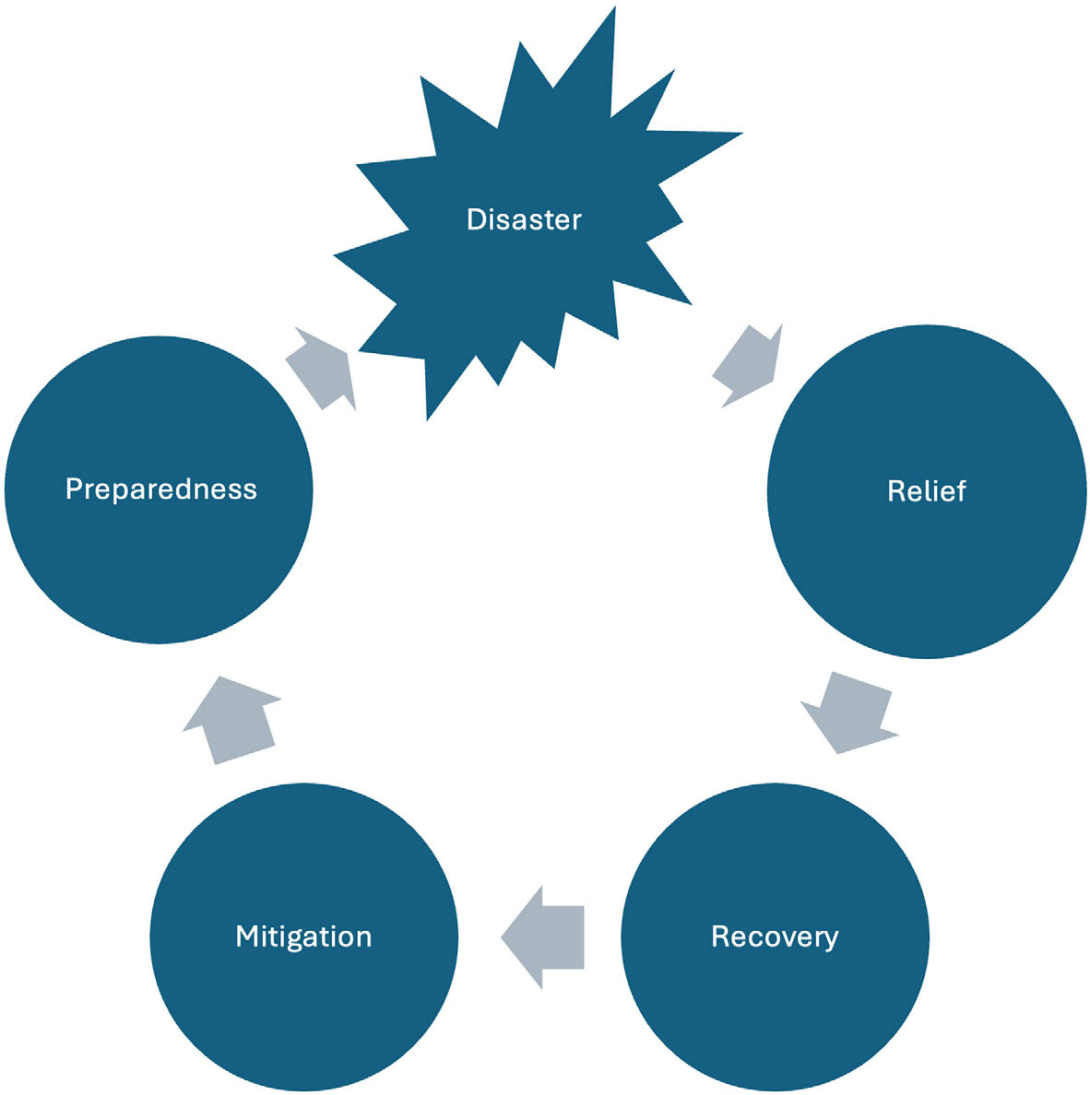
Disaster management cycle **Source:** authors, based on Asian Disaster Reduction Center (2005, p. 14).

In disaster management, a well‐known model of the ‘cycle of disaster management’ identifies stages after a disaster (such as relief, recovery, and rehabilitation) followed by stages before a disaster (such as prevention, mitigation, and preparedness) (see Figure 1) (Arifah, Mohd Tariq, and Juni, 2020; Balamir, 2023). The model has been in use for many decades, but has long been criticised, among other things for being too neat—there are rarely specific ‘phases’ in disasters, although responses are organised according to this understanding. It is well documented across the literature that disasters resulting from natural hazards can, for example, develop into extended humanitarian crises or lead to disruptive economic or political instability and tension (Siddiqi, 2018; Olson, 2000; Desportes and Moyo‐Nyoni, 2022). Temporally, this can play out as a slow‐onset event with prolonged aftereffects, or present chronic adversity (Nixon, 2011), further complicating the phases of the disaster management cycle. Another criticism is its linear understanding—yet it is known that following a disaster, initial euphoria may be met by increased depression and other psychosocial impacts, even some years later (Cretney, 2018).

In Australia and elsewhere, the increasing frequency and severity of disasters means that there may be little, if any, time for recovery. In the north of the state of NSW alone, three floods in quick succession in 2022, which themselves followed the devastating bushfires of 2019–20, have rendered thousands of people homeless and in need of protracted relief. The term recovery—and the cycle of disaster—also assumes rather mechanistically that recovery happens and will then come to an end. For many, of course, it does; but for others, the emotional, psychological, physical, and financial scars left by a disaster may take decades to heal, if they do at all. What is missing, then, from examinations of disaster recovery is a fuller understanding of the disaster–recovery nexus, one which acknowledges the important stages preceding a disaster and accommodates the varied and very localised aspects of what it means to recover from a disaster and reconstruct the unique aspect of community in its wake.

Internationally, disaster recovery is pursued by many service and humanitarian organisations as a means to support recovery and reconstruction after a disaster. Moreover, such organisations play a central role in local disaster planning, prevention, mitigation, and preparedness initiatives (Hollis, 2015). For all the positive work that service and humanitarian organisations provide, there are numerous examples of how they contribute to the poor management of disasters. For instance, after the 2010 earthquake in Haiti, ‘local authorities. .. complained that three months after the earthquake they felt “like strangers in [their] own city”’ (Grünewald and Binder, 2011, p. 43). In part, this situation can result from local authorities being overwhelmed and not adequately resourced to contend with the challenges posed by disasters. This can be worsened by the tendency for regional and international responding organisations to neglect understanding of the dynamics of local communities (Heffernan et al., 2024) and to overlook local stakeholders in relief and recovery operations (Sanderson, 2019, p. 60).

In recent years in Australia there has been considerable investment in disaster management, primarily in response to the 2019–20 bushfires, including at the federal level with the formation of the National Emergency Management Agency (NEMA). In NSW, the Office of Emergency Management (OEM) was reinvented as Resilience NSW in 2020 with a remit to support disaster recovery; in 2022, it was abolished and replaced with the NSW Reconstruction Authority (NSWRA). The demise of Resilience NSW can be attributed in part to a lack of clarity on what stages of the disaster cycle the new entity was supposed to address. It was set up by the Department of Premier and Cabinet with a mandate to support recovery and build resilience. With leadership provided by an executive with strong disaster response credentials, the organisation struggled to find its place, being strongly criticised for its perceived inactions following the 2022 northern rivers floods. When the state government changed in 2022, Resilience NSW was dissolved and replaced by the NSWRA, with a stronger focus on preparedness, mitigation, and recovery. Its website states that the organisation ‘aims to take a more focused approach to recovery, reconstruction and in particular mitigation and adaptation (NSW Reconstruction Authority, 2024).

#### 1.1.2 Community‐centred approaches to recovery

Among the varying discourses and disagreements within disaster management, and as previously noted, there is long‐standing agreement that the most effective recovery is community‐centred.^2^ This extends to practitioners, policy, and in research. Citing numerous sources, Gibbs et al. (2021, p. 4) note that such approaches ‘are regarded as the optimal approach to sustainable disaster recovery, fostering self‐reliance and self‐determination’. The *NSW Recovery Plan* of 2021 asserts that ‘[s]uccessful recovery is community‐centred, responsive and flexible, engaging with community and supporting them to move forward’ (Resilience NSW, 2021, p. 4). In this way, community‐centred approaches promote the dual strengths of supporting communities to participate substantively in their recovery, while also creating the space for local communities to become recovery decision‐makers (Vallance, 2015). The *Inquiry into Western Australia's Natural Disaster Relief Arrangements* recorded one respondent's views: ‘Above all, the recovery worked as well as it did because it was based on the idea that local community members are the best ones to take on the responsibility to help and have the confidence of the affected community, with the local Recovery Committee being supported by the government and non‐government organisations’ (Community Development and Justice Standing Committee, 2007, pp. 71–72).

Communities of course can be massively diverse, range from cohesive to fractured, and be constantly changing. As Owen (2018, p. 68) records in an interview with a government official, ‘[t]here's a sort of a myth that communities are cohesive, that they have, if you like, a shared perspective. Often communities are quite fractured [before a disaster] but you don't notice it because people just get on separately doing their own thing’.

Owen's (2018) research into community‐centred recovery concludes that ‘participant responses fell into one of two viewpoints. One was that communities did not necessarily have the capacity, knowledge or skills to lead the recovery process, at least initially, because of disruption and trauma. … The other view was that communities were the obvious leaders of recovery from the moment of the emergency event. Community participants in particular said that community members and groups are usually the first responders and gave examples of the processes and activities communities put in place to support recovery from the outset’ (Owen, 2018, p. 67). For this reason, community‐centred recovery can be plagued by existing tensions or provide ‘opportunities to challenge existing social divisions and inequalities and to promote democratic social change’ (Crawford and Morrison, 2021, p. 549). This is, of course, hardly surprising. A disaster impacting a community usually exposes pre‐existing conditions, both positive and negative.

Community‐centred recovery does not mean that communities are left to fend for themselves after a disaster (although this happens). It also does not mean that external support is not required—the definition of a disaster, after all, is an overwhelming event for which outside assistance is needed. Emergency services, including fire and police departments, local, state, and national governments, NGOs and voluntary organisations, and a range of others, all offer vital assistance to people caught up in fires, floods, and other emergencies. In the immediacy of an impending fire or flood, it is commonplace to issue agency/government‐led evacuation orders and other life‐saving edicts and actions. Yet, it may be hard for agencies organised as top‐down command‐and‐control organisations to then in effect cede power to communities—this is discussed later.

#### 1.1.3 Participation in decision‐making

**FIGURE 2:**
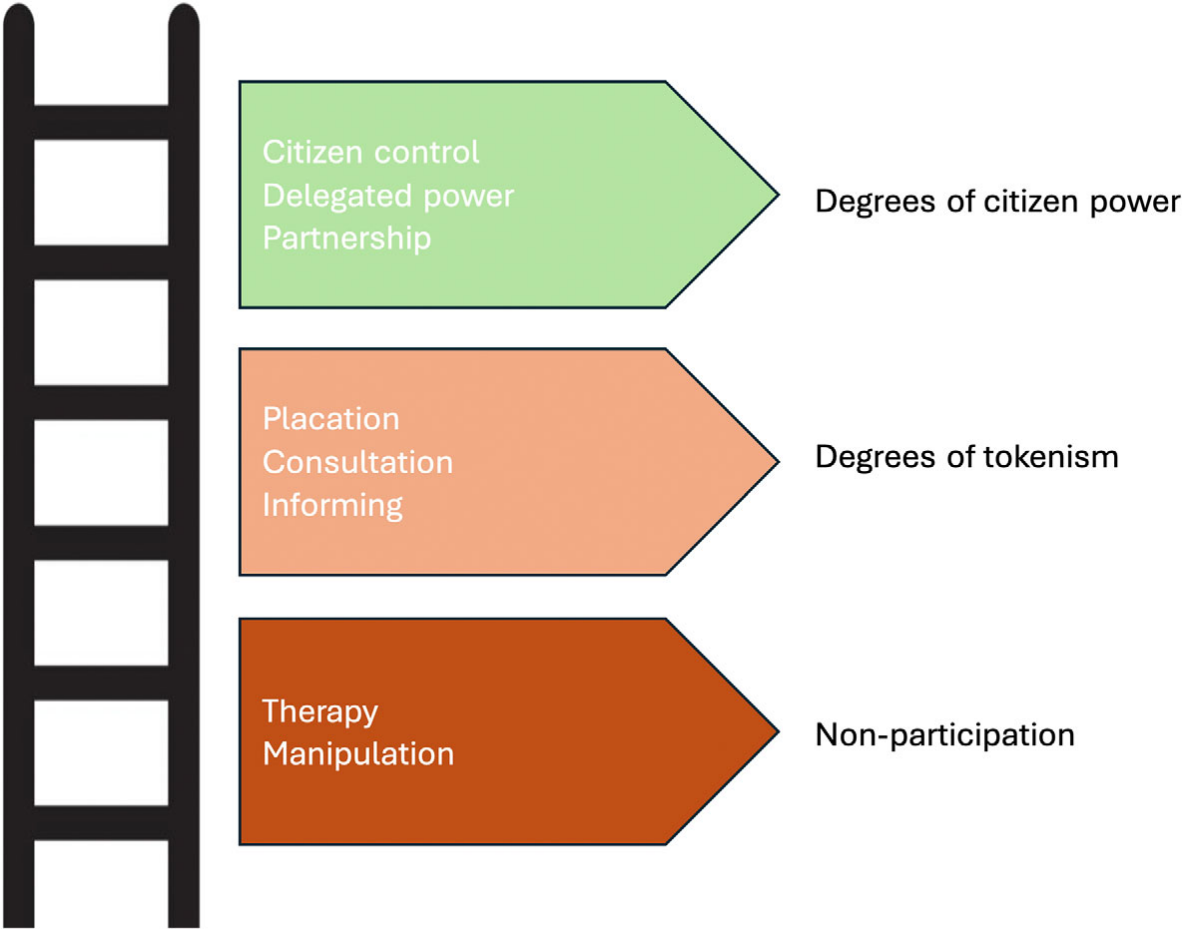
Arnstein's Ladder of Citizen Participation **Source:** authors, based on Arnstein (1969, p. 217).

Implicit in our definition of community‐centred recovery—that is, ‘approaches that mobilise assets within communities, encourage equity and social connectedness and increase people's control over their lives’—is that communities play a central role in decisions that affect them. The key feature of a ‘community‐centred approach’ is the degree of participation of community members in decisions that affect them. There is a large body of literature from many sources, such as international development and urban planning, that has explored participation in decision‐making (see, for example, Chambers, 1997; Anderson, Brown, and Jean, 2012). Perhaps one of the best known and long‐standing measurements of citizen participation that has stood the test of time is Arnstein's (1969) Ladder of Citizen Participation (see Figure 2). Developed in the United States, Arnstein's ladder identifies eight ‘rungs’ within three groups: ‘non‐participation’ at the bottom, comprising manipulation and therapy; ‘degrees of tokenism’ in the middle, encompassing informing, consultation, and placation; and ‘degrees of citizen power’ at the top, incorporating partnership, delegated power, and, at the summit, citizen control.

Within the international literature on humanitarian response, there is considerable research into how to get to the top of the ladder (Scott‐Smith, 2018). Among this is work on the concept of ‘partnership’, a widely adopted approach wherein ‘more powerful’ agencies and authorities can productively engage with ‘less powerful’ entities such as community‐based organisations and civil society groups. Power here is taken to mean mandate, budget, and/or regulatory control. While there are some good examples of partnership, these are typically hard to come by. The International Federation of Red Cross and Red Crescent Societies' *World Disasters Report* of 2016, for instance, asks whether ‘partnership’ is the most ‘abused word’ in disaster response (IFRC, 2016, p. 169). Problems in achieving successful partnerships include power asymmetries, conflicting priorities, differing time frames, and budgets. What is more, ‘powerful agencies’ often position local communities as passive ‘partners’ in formal disaster recovery initiatives (Heffernan et al., 2024), thereby perpetuating a cycle of development premised on an imbalance of power.

#### 1.1.4 How good are agencies at community participation in recovery?

This paper argues that, across the board, the answer to this question is ‘could do better’. That is not because of any covert agendas of agencies, but rather, that the systems and tools currently used do not lend themselves to genuine community‐centred recovery, at least in Arnstein's (1969) sense of degrees of citizen power. There is plenty of evidence for this, in Australia and elsewhere. Internationally, a frequent finding of disaster recovery evaluations is that insufficient attention was given to local priorities. Following the 2015 earthquakes in Nepal, a survey found that ‘[w]hen women were asked if their particular problems are being addressed, a resounding 73% said “very little” or “not at all”’ (Sanderson et al., 2015, p. 5). After the 2011 earthquake in Christchurch, New Zealand, Cretney (2018) found widespread dissatisfaction among people regarding their level of engagement. She notes one interviewee as stating: ‘There seems to be a façade of asking people's opinions and taking people's ideas on board. But it's just that. They don't actually listen’ (Cretney, 2018, p. 125). She adds that ‘[o]thers described the predetermined nature of decisions and the use of participatory processes to “rubber stamp” decisions’ (Cretney, 2018, p. 125). Cretney (2018, p. 125) concludes that ‘[t]he response of participants to this research indicated that many residents in Christchurch did not have a positive experience of the official and formal processes for engagement with the disaster recovery plans and initiatives’.

Some of the most compelling evidence comes from Anderson, Brown, and Jean's (2012) five‐year study of people's experiences of operational agencies following a disaster. Close to 5,000 people were interviewed from across the world. The key finding was that people felt that they were not listened to. One quote from Myanmar summarises a common experience: ‘There are times that NGOs do not provide what people really need, the projects come from above, top‐down. They should listen to the people from the communities’ (Anderson, Brown, and Jean, 2012, p. 39). As part of our online survey carried out in Australia (discussed further below), when asked about how their voices were heard, one respondent emphasised: ‘Voices of people [in recovery decision‐making] were not included. Only certain community members appeared to have the “right” to be making decisions’. Another stated: ‘Services have just come in here. We're here to rescue you. And here we've got this, this, and this, like, well, we don't actually need that. Have you actually asked us what we've already got?’

### 1.2 Part two: case study—community‐centred disaster recovery in NSW

For this paper, we undertook research into the experiences of communities affected primarily by the 2019–20 bushfires that struck NSW. As the Report of the Royal Commission into National Natural Disaster Arrangements (2020, p. 5) describes, ‘the fires started in Australia's hottest and driest year on record, with much of the country that burnt already impacted by drought. … Over 24 million hectares were burnt. Many Australians were impacted, directly or indirectly, by the fires. Tragically, 33 people died and extensive smoke coverage across much of eastern Australia may have caused many more deaths. Over 3,000 homes were destroyed. Estimates of the national financial impacts are over $10 billion. Nearly three billion animals were killed or displaced and many threatened species and other ecological communities were extensively harmed’. The government responded with approximately AUD 280 million in funding. Emergency recovery centres were mobilised, with support provided to local councils. As Heffernan et al. (2024, p. 5) point out: ‘Local and state government agencies and NGOs were the main recovery actors. Physical recovery, over and above social recovery, was pursued. Government‐centred and NGO‐supported assistance illustrates the mainstream approach to disaster management in Australia, with state and emergency actors “steering” and NGOs and communities “rowing”’.

#### 1.2.1 Research approach

A mixed‐methods approach (Johnson, Onwuegbuzie, and Turner, 2007; Creswell and Plano Clark, 2018) was adopted to strengthen the accuracy of our findings, utilising a concurrent triangulation design (Creswell et al., 2003; Bryman, 2006). We held key informant (KI) interviews with and administered an online survey to those who had experienced disaster recovery in Australia within the past five years. For qualitative data, we interviewed 14 KIs who had had recent experience of disaster recovery, during the 2019–20 fires, as well as elsewhere in Australia. KIs were practitioners from local, state, and national agencies, NGOs, and emergency services (see Table 1). Their work experience ranged from 2–20 years. KIs were asked nine questions (designed to structure a conversation) relating to their perceptions of recovery, what community‐centred recovery means to them, good and bad examples of community‐centred recovery, and how community‐centred recovery could be better supported. Interviews lasted for an average of 30 minutes and were recorded with permission, after participants were assured of anonymity and that they were not representing their agencies.^3^

**TABLE 1:** Key informants.

Organisation type	Male	Female
NGOs	1	3
State operational agency	1	3
Federal agency	1	–
Local government	–	3
Emergency services	2	–

**Source:** authors.

The themes that materialised from the analysis of the semi‐structured interviews were then crosschecked with the data that emerged from the quantitative analysis. An anonymous online survey, open to people from across Australia, was conducted among community members who were 18 years or over and had experienced a disaster event (in particular a flood and/or a fire) over the past five years. In total, 168 responses were collected. Sixty‐six per cent identified as ‘woman/female’ and 29 per cent identified as ‘man/male’. One per cent identified as ‘non‐binary’ and four per cent preferred not to say. Thirty‐five per cent of respondents were aged between 55 and 64 and 30 per cent were aged between 45 and 54. Eighteen per cent were aged between 35 and 44 and seven per cent were aged between 25 and 34. Nine per cent were aged over 65 and just one per cent were aged between 18 and 24. Seventy‐eight per cent of respondents had spent more than five years in their location, with the remainder (22 per cent) being in residence for less than five years. Ninety per cent of respondents identified not as Aboriginal and/or Torres Strait Islanders, while five per cent did identify as such (and five per cent preferred not to say). We would of course have preferred more people aged between 18 and 24 years to respond, as well as more people identifying as Aboriginal and/or Torres Strait Islanders.

The survey asked participants about their experiences of responding agencies, such as NGOs, emergency services (police and fire departments), and local and state government bodies. Questions related to: the effectiveness and timeliness of agency activities; whether people felt that they were consulted adequately; whether they thought that their voices were heard; whether community members were treated equally; whether recovery efforts were community‐centred; and whether the recovery was a success overall. Participants were asked to respond to statements using a five‐point scale ranging from ‘strongly agree’ to ‘strongly disagree’, with ‘neutral’ also as an option. At the end of the survey, respondents were invited to write down any comments they felt were important to make.

The mixed‐methods approach not only allowed us to obtain richer data through a larger sample size, but it also led to a more exploratory analysis of the phenomenon by comparing the different perspectives of emergency service practitioners and community members.

#### 1.2.2 Findings

We asked participants in the KI interviews what community‐centred recovery meant to them. One answered: ‘what it means is the community taking charge and taking control of their destiny and where they end up’ (KI2). Another remarked: ‘what that means for me is I feel that those that are most impacted by the emergency should be able to shape their recovery. … To enable them, I guess to take a bit of a control around getting back to some sort of normality’ (KI1). A third said: ‘It's important that there are both government and non‐government organisations and agencies walking alongside communities, and I guess not necessarily guiding them—but just being there to support them’ (KI2).

Communities, however, as previously noted, are rarely homogeneous. In international development there is extensive research on the concept of community and the loaded assumptions of a homogeneous grouping that the term ‘community’ can invoke. Communities, of course, can have competing interests and priorities, with people themselves ranging from constructive ‘local champions’ to those who can be inconsistent, fickle, and self‐interested. When asked what their view of community‐centred recovery is, one experienced responder stated: ‘Probably 10 to 13 different communities of interest across our Shire and that means different things to different people depending on the makeup of that community, the demographic, the socioeconomic profile’ (KI2).

As another KI from local government explained: ‘The community I'm working with, they are a community that wanted to be together. They'll be most, I would say, impacted out of the bushfires. So they've come together to build a community centre. They really came together because to have a lot of ex‐public service people in there who are pushing this community. If you go across the road, I've got another community [that] came to me and said, “we're actually not even a community yet. What can you do to help support us to build that community up?”’ (KI4).

**FIGURE 3:**
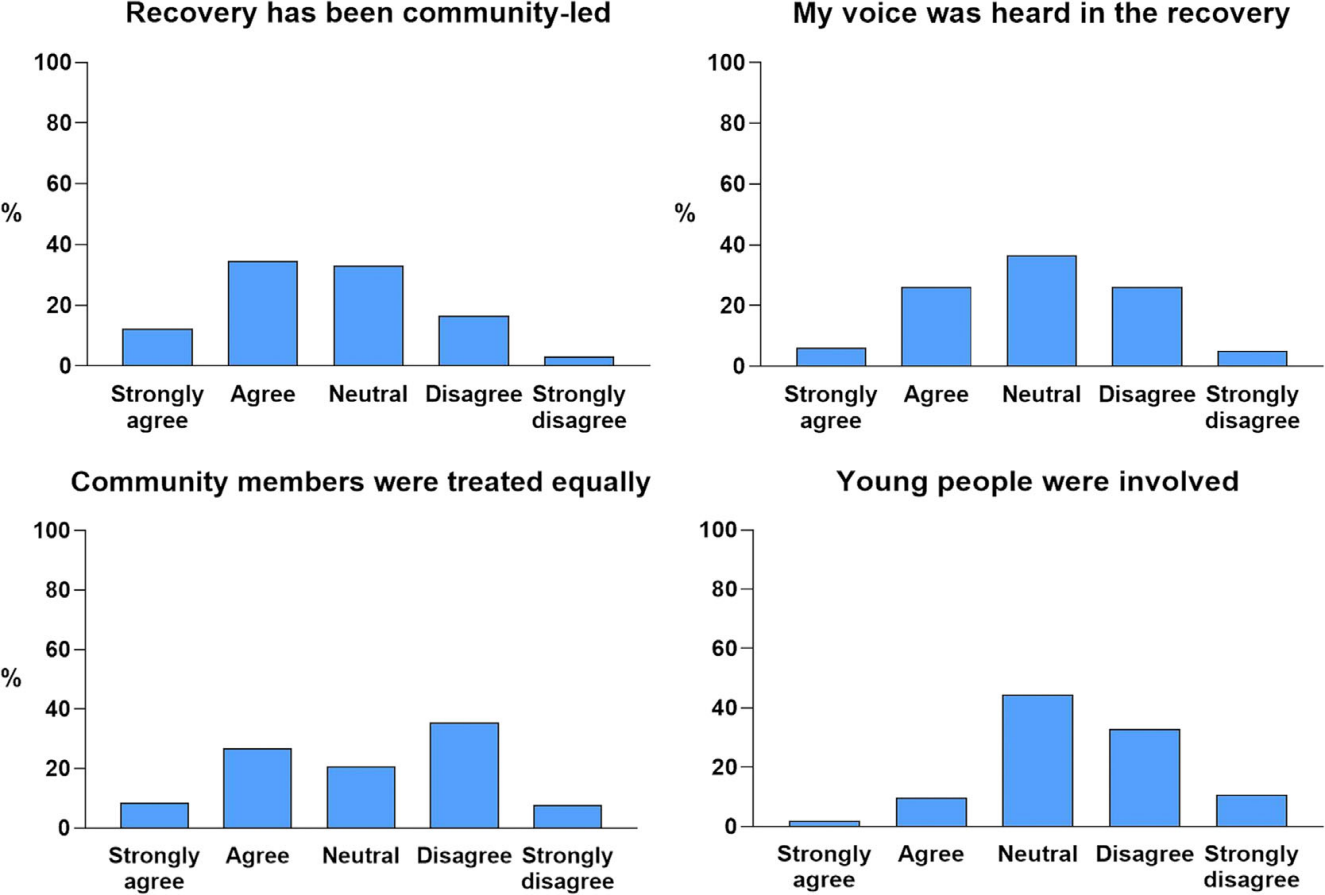
Survey findings. **Source:** authors.

The findings of our online survey (see Figure 3) of how people perceive the reality of community‐centred recovery, their voice, and the voice of others, suggest that it would be difficult to conclude that the responses equate to climbing to the top rungs of Arnstein's (1969) ladder (see Figure 2). In response to the statement, ‘Recovery has been community‐led’, slightly more than one‐half (52 per cent) of respondents agreed or strongly agreed, 38 per cent were neutral, and the remainder disagreed or strongly disagreed. Two‐thirds of respondents could not agree with the statement, ‘My voice was heard in the recovery’—only around one‐third (32 per cent) agreed or strongly agreed. Around the same proportion disagreed or strongly disagreed (30 per cent), while 38 per cent responded as neutral. Nearly one‐half of respondents (48 per cent) disagreed or strongly disagreed with the statement that ‘Community members were treated equally’; only 35 per cent agreed or strongly agreed, with the remainder being neutral. Only a small number of respondents (11 per cent) agreed or strongly agreed that ‘Young people were involved’; close to one‐half (48 per cent) disagreed or strongly disagreed.

### 1.3 Part three: challenges to supporting community‐centred recovery

While held up as the gold standard, our data demonstrate that community‐centred recovery is challenging for several reasons. Two of the most notable, we argue, are the command‐and‐control culture of emergency authorities and the resultant agency key performance indicators (KPIs).

#### 1.3.1 Command‐and‐control culture applied to recovery—relief is the enemy of recovery

A well‐known phrase in humanitarian disaster response is that ‘relief is the enemy of recovery’. That is, continuing to do things for communities can eliminate communities' own efforts geared at recovery. While, as noted earlier, a strongly organised, clear system is undoubtedly needed in the immediate wake of a disaster to coordinate the initial response, we reviewed for this research many positive examples of work by responding agencies that saved lives and prevented properties from being destroyed. Especially noteworthy was the timely information given by such agencies on when to evacuate.

However, if emergency response continues into the recovery phase, then there is a risk of a power imbalance continuing, with communities becoming disenfranchised, not consulted with, and ignored. As a community member observed following the agency response to the 2016 bushfires in Waroona, ‘there seems to be a them and us situation and a feeling that we the local community are being dictated to by the different departments which causes some anger amongst those affected’ (Waroona Bushfire Special Inquiry, 2016, p. 244). The same person also stated that ‘[t]here needs to be a consultation process between those who can help in the community and the fire controllers so that we can get the best possible outcome in these situations’ (Waroona Bushfire Special Inquiry, 2016, p. 244).

The systems, approaches, and cultures used in the response were sucked into recovery—an analogy with healthcare would be that accident and emergency is put in charge of community healthcare. Command and control also, by necessity, centralises and tightly controls power. This is the antithesis of community‐centred recovery, including local councils versus state and federal authorities. The quote provided earlier in the Haiti example of local councils feeling as though they were ‘strangers’ in their own cities also plays out in Australia and elsewhere. As one KI from local government noted in discussing the Black Summer fires of 2019–20: ‘They [the responding agencies] weren't looking at the better interests of the community. They were looking at their policies, their procedures, their guidelines, their outputs.. .. I've seen it work the worst when there hasn't been collaboration with services on the ground and where there was no consultation with the community’ (KI2).

#### 1.3.2 Agency KPIs

Agency KPIs that are aligned towards measures of agency success can get in the way of community priorities. KPIs favour simplicity, clarity, and ‘results’ that are tangible, measurable, and predictable. By and large, they do not like a ‘messy reality’, serendipity, or imprecision, when in fact this is par for the course in community recovery. KPIs are often aligned to inputs and outputs, but rarely outcomes. Outcomes are seldom measured as they occur months and years after an event, which is outside the time frame and budget of project‐based approaches. Rather, KPIs are frequently aligned with, and measured using, budget expenditure according to a fixed time frame. In plenty of recovery operations the pressure is on to spend a lot in a short amount of time. If this is the case, a form of short‐termism can dominate, and when it does, KPIs can also become public optics of spending money in service of recovery, albeit with little to no community consultation.

#### 1.3.3 Agency time frames

A further issue is agency time frames. As noted earlier concerning the cycle of disaster, response structures are often organised around tight time frames. While there is an argument that recovery operations need to be finite (not least for budgetary reasons), recovery realities often depict a different story. Agencies are rarely rewarded for investing in long‐term measures that may exceed their project timelines, and the mandated time scales of their political masters.

An example is the provision of temporary shelter after a disaster, which very often does very little for long‐term recovery, given its high cost and short life span. In Australia, the provision of short‐term ‘pod villages’ to those affected by the 2022 northern rivers floods in NSW is currently a matter of tension. While there can be some benefit in supplying temporary homes—one located on the property of a destroyed house allows the homeowner to live somewhere while rebuilding—there are several challenges when developing smaller enclaves, or villages. These include the very high unit cost (when infrastructure and services are included), their temporary nature and what happens to tenants when leases expire, securing temporary use of land that the owner will want back later, and reported conflict and antisocial behaviour. Perhaps the biggest challenge is what happens after living in pod villages? Many people have nowhere to go, and authorities are reluctant to enact evictions. At the time of writing there are few indications of how the government intends to demobilise the villages. In the second half of 2023, only 75 households were recorded as having exited temporary arrangements, more than 18 months on from the initial flooding event.

#### 1.3.4 Choosing how to engage

**FIGURE 4:**
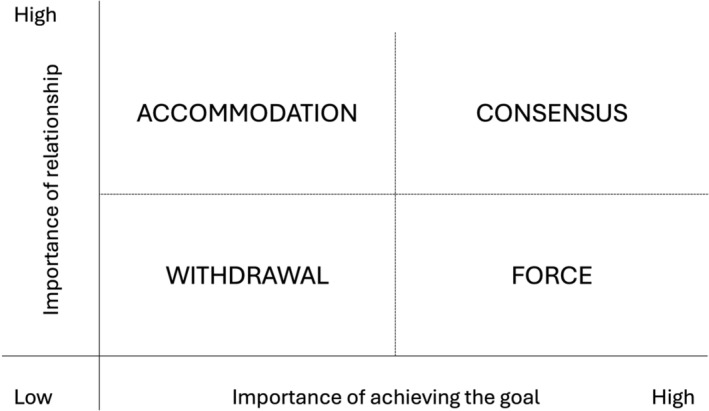
Importance of achieving the goal and importance of relationship **Source:** authors based on Coburn (n.d.).

This paper has discussed the tension regarding command‐and‐control response extended into recovery, and how this can promote a ‘them and us’ culture that disenfranchises communities. But a command‐and‐control approach is an agency‐ and culture‐based decision, not an automatic given. There are choices when it comes to how agencies, and agency members, can work. Figure 4, based on the work of Coburn (n.d.), illustrates the choices there are for collaboration, and the responses they evoke. The horizontal axis shows the ‘importance of achieving the goal’ while the vertical axis shows the ‘importance of relationship’. Our current systems and approaches for recovery incentivise agencies towards ‘force’, when achieving the goal is high and where valuing the relationship is low. Ideally, of course, ‘consensus’ would be the favoured outcome, but this takes time, is much harder, and involves actors (state and local government, NGOs, communities, and others) negotiating power imbalances, competing interests, and differing mandates.

The comments of several KIs interviewed for this paper serve to illustrate this point. As one experienced KI noted, ‘if it's around the recovery work, what we need is humanitarians, we don't need [combat agencies]’ (KI3). According to another, ‘[t]here was no collaboration. There was no consultation and where the locals’ needs were not heard … they didn't actually do anything with that consultation with the tick flick exercise’ (KI3). Another KI describing the responses of two large NGOs stated that ‘[m]y biggest problem over the community recovery programme is external forces. [They] come in, to tell us what we need and with no flexibility’ (KI4).

Cretney (2018, p. 123) identifies the better outcomes that are achieved when participation is included in disaster recovery: ‘an analysis of two communities in the United States during recovery from floods … found that the local administration that engaged the community in decision making was “more likely to believe that citizens had an effect on decisions made and that the city made attempts to involve them”. Other studies on participation and recovery have reported improvements in the success of post‐disaster initiatives, an increased trust in authorities and assistance in the psychological processing of the disaster experience’.

### 1.4 Discussion: a call to change

If we want genuine community‐centred recovery (and we all seem to agree that we do), then change is needed in how disaster recovery is framed and understood. There are several areas to consider; we have not discussed in this paper, for instance, that devolving more power to local authorities for stronger community‐level engagement is a missed opportunity. Three areas relating to what we have considered, however, are (i) the need to include in recovery the underlying causes of vulnerability, (ii) to measure success differently, and (iii) to change the narrative on who ‘owns’ disasters.

#### 1.4.1 Include in recovery the underlying causes of vulnerability

Counter‐intuitively perhaps, too much focus is given in recovery to the disaster itself, rather than to the underlying vulnerabilities that made the event so bad. Disasters are defined as natural hazards, such as a flood, that impact on vulnerable circumstances, such as people's homes situated on a floodplain. Of course, immediate help is needed for relief operations. Yet, if the focus on the disaster perpetuates, the underlying issues that worsened the disaster, such as the location of homes, which made people vulnerable in the first place, tend to be ignored in recovery efforts. As news outlets noted regarding the northern rivers floods of 2022, the flood impacted on a housing crisis disaster (MacKenzie, 2022), referring to the pre‐existing shortage and cost of local housing. Worse still, government responses may only focus on one disaster at a time. In NSW, recovery efforts following the 2019–20 bushfires were under way when the 2022 floods struck. On several occasions, bushfire funding rules prevented organisations from using those resources for flood response, despite working in the same communities. Greater flexibility and catering for the unexpected are required for such occasions. There is substantial research from across the world on how to improve this situation, but adoption in existing (power) structures is often a problem. Difficulties include working with and through local actors, structures, and networks, utilising and expanding preparedness initiatives that have already been adopted locally, focusing on local capacity‐building as a vital form of assistance and support, tailoring the coordination of disaster management and resilience‐building, and recognising the regional nature of the response (Sanderson and Ramalingam, 2015; Paul, Acharya, and Ghimire, 2017).

#### 1.4.2 Measure success differently—process over product

A perennial challenge for effective recovery lies in how ‘success’ is measured. Whose success? As noted, too often success relates to political measures of success, which are tightly time‐bound by political cycles and ‘being seen to do something’. Success is too frequently viewed through the eyes of the providers. The current narrative is that successful recovery is situated largely in the provision of short‐term fixes. As discussed earlier, providing temporary shelter after a disaster, such as pod villages, is expensive and only delays solving the problem to a later date (while creating new problems); this represents an approach that looks like a ‘solution’ has been found, kicking the problem down the road.

Measuring processes over products may seem unsatisfactory in terms of saviour‐oriented optics, but the reality is that recovery can take years, decades, or even a lifetime. Incremental improvements that are rooted in the messiness of real life (see the point above) are probably much better suited to supporting community recovery. Approaches that purposefully seek out and capitalise on serendipity, opportunism, and imperfection have been employed in international disaster recovery and in international development for decades. Action planning, which relies on catalysing communities to enact their priorities, and where agency ‘providers’ become more effective as ‘supporters’, is one such approach (Hamdi and Goethert, 1997). Action planning, though, reverses many of the assumptions to which current recovery mechanisms adhere and that have been discussed in this paper, such as prioritising process over product, engaging in starting points, such as nurturing community priorities rather than imposing ‘solutions’ and devolving power to as local a level as possible, and fixing time frames in accordance with people's realities rather than political ones.

#### 1.4.3 Change the narrative on who ‘owns’ disasters—remove the ‘them and us’

In Australia, as elsewhere, there is a perception that disasters are ‘owned’ by the emergency services. The media usually focuses on dramatic footage of post‐disaster rescue while interviews on what should be done are invariably undertaken with officials in uniforms. While it is no surprise that the media reports on dramatic situations, the issue runs deeper. Structurally, the police force (at least in NSW) oversees disaster response, while other emergency services, such as the NSW Rural Fire Service and the NSW State Emergency Service, make decisions that strongly affect communities and how they recover. The risk, as discussed throughout this paper, is that power stays with the authoritarian structure into the recovery phase and is insufficiently ceded to include communities. It is important to reiterate that emergency services play a central, vital, and much‐needed role in the immediate response, but they should assume a quickly diminishing part in recovery. As our research, and that of others, finds, top‐down command‐and‐control structures are ill‐suited to include (let alone prioritise) bottom‐up community‐centred recovery. An earlier quote from a community member referred to the ‘them and us’ culture, between community members and responding agencies. To change this, choices must be made in how agencies choose to respond, and the continuation of command and control into recovery must not be a given.

## ETHICS STATEMENT

This paper reports analysis of primary data. The ethics of data collection and analysis were approved by the University of New South Wales.

## Data Availability

The data that support the findings of this study are available from the corresponding author upon reasonable request.

## Endnotes

^1^
This definition is closely based on that provided by the Government of the United Kingdom of community‐centred public health. For more information, see https://www.gov.uk/government/publications/community-centred-public-health-taking-a-whole-system-approach (last accessed on 6 August 2024).

^2^

Dictionary.com defines a community as ‘a social group whose members have something in common, such as a shared government, geographic location, culture, or heritage’. In addition, it ‘can also refer to the physical location where such a group lives’. For more information, see https://www.dictionary.com/browse/community (last accessed on 15 July 2024).

^3^
This research received ethics clearance (no. HC210846) as part of a project of the Resilient Towns Initiative, funded by Regional NSW. The research conducted for this paper was not funded by the project.
